# An assay to measure poly(ADP ribose) glycohydrolase (PARG) activity in cells

**DOI:** 10.12688/f1000research.8463.2

**Published:** 2016-09-15

**Authors:** Dominic I. James, Stephen Durant, Kay Eckersley, Emma Fairweather, Louise A. Griffiths, Nicola Hamilton, Paul Kelly, Mark O'Connor, Kerry Shea, Ian D. Waddell, Donald J. Ogilvie

**Affiliations:** 1 Drug Discovery Unit, Cancer Research UK Manchester Institute, University of Manchester, Manchester, UK; 2 Oncology iMED, AstraZeneca Pharmaceuticals, Macclesfield, UK

**Keywords:** PARG, PARP, olaparib, DNA damage response, Base excision repair, MMS, ADP ribosylation

## Abstract

After a DNA damage signal multiple polymers of ADP ribose attached to poly(ADP) ribose (PAR) polymerases (PARPs) are broken down by the enzyme poly(ADP) ribose glycohydrolase (PARG). Inhibition of PARG leads to a failure of DNA repair and small molecule inhibition of PARG has been a goal for many years. To determine whether biochemical inhibitors of PARG are active in cells we have designed an immunofluorescence assay to detect nuclear PAR after DNA damage. This 384-well assay is suitable for medium throughput high-content screening and can detect cell-permeable inhibitors of PARG from nM to µM potency. In addition, the assay has been shown to work in murine cells and in a variety of human cancer cells. Furthermore, the assay is suitable for detecting the DNA damage response induced by treatment with temozolomide and methylmethane sulfonate (MMS). Lastly, the assay has been shown to be robust over a period of several years.

## Introduction

Cells use a varied array of post-translational protein modifications to regulate signalling pathways. One of these is ADP ribosylation whereby single units or multiple, branched polymers of ADP are covalently attached to a target protein. For example, poly(ADP) ribosylation (PARylation) plays a particularly important role in base excision repair with poly(ADP) ribose (PAR) polymerase 1 (PARP1) detecting single strand breaks that occur during this pathway. PARP1, which binds to these single strand breaks undergoes auto-modification creating up to 200 PAR chains ^1^, that subsequently recruit the rest of the repair machinery including XRCC1 and POLB to complete the repair. The ADP ribose chains on PARP are hydrolysed by the enzyme poly(ADP) glycohydrolase (PARG). The correct functioning of this pathway is key for repair to complete. Cancer cells rely on DNA repair more heavily than normal cells and inhibitors of these pathways have been in preclinical and clinical evaluation for a number of years ^2^. The success of this strategy is exemplified by the inhibition of PARPs using olaparib that recently gained regulatory approval for use in ovarian cancers ^3^. However, as auto-modified PARP1 is less able to bind DNA, inhibition of PARG has also been hypothesized as a suitable therapeutic target. This is even more germane as there are now 17 known members of the PARP (otherwise known as ADP ribosyl transferase diptheria-like; ARTD) family yet no known close homologues of PARG. PARG inhibition may therefore offer a more direct approach to derailing the DNA repair pathway without the problems of redundancy. Molecules that are claimed to inhibit PARG have existed for some time. Many of these are large tannin-like molecules such as gallotannin which have been shown to have a number of effects unrelated to PARG inhibition (e.g. anti-oxidant properties ^4^). Other compounds, such as APD-HPD and rhodamine-based PARG inhibitors (RBPIs), have shown good specificity for eukaryotic PARG but are either not cell permeable or have only been tested in biochemical assays ^5– 7^. Attempts to discover new synthetic PARG inhibitors have resulted in compounds that also inhibit PARP or have low potency ^8– 10^. We therefore carried out a high throughput screen (HTS) directed against human PARG and identified a small number of hits which were carried through to a computational and medicinal chemistry programme ^11^. We were mindful of the need to develop assays to detect cell-permeable inhibitors and the method development is contained herein.

## Materials and methods

### Cell culture and materials

Unless otherwise stated, all reagents were purchased from Sigma-Aldrich (Dorset, UK). Methylmethanesulfonate (MMS) was diluted in dimethyl sulfoxide (DMSO) to 250 mg/mL from the purchased stock. Temozolomide was dissolved in DMSO at 20 mg/mL. All cells were purchased from ATCC (LGC, Teddington, UK) unless otherwise stated and regularly checked for mycoplasma and were regularly sent for authentication. HeLa cells were maintained in RPMI 1640 (Sigma R0883) + 1% Glutamax + 10% FBS. PARG KD cells were purchased from Tebu-bio (PARG Hela Silencix 01-00085, Peterborough, UK) and maintained in DMEM + 1% Glutamax + 10% FBS + 125 µg/mL hygromycin B (#10687010; ThermoFisher, Northumberland, UK). All cells were maintained at sub-confluence at 37°C in a humidified incubator containing 5% CO _2_ in the absence of antibiotics. Mouse embryonic fibroblasts were cultured in DMEM (# 10938-025; Invitrogen, Paisley, UK) + 10% FBS + 1% L-glutamine and H1048 cells were grown in RPMI (# 21875-034; Invitrogen) + 10% FBS. SW620 cells were cultured in DMEM (#D6546) + 10% FBS + 1% L-glutamine. Dose response curves were generated using Prism v5.2 (Graphpad Software Inc, La Jolla, USA).

### PAR chain assay and nuclear count

Exponentially growing HeLa cells were trypsinized and resuspended in complete media before being filtered through a 40 µM cell strainer (#352340, BD Falcon, Oxford, UK). Cells were then counted using a Muse cell counter (Merck Millipore, Hertfordshire, UK) and seeded in 30 µL of media at 4×10 ^4^ cells/mL in Greiner 384-well plates (#781091, Greiner Bio-One, Stonehouse, UK) and placed in a cell culture incubator. After 16–24 h the plates were centrifuged briefly at 164× *g* and the cells dosed with compound(s) or vehicle (DMSO) control using an Echo 550 (Labcyte, Dublin, Ireland). Initially an 8-point dose response with two replicates per point was used with doubling dilutions (0.02–30 µM) and this was extended to a 10-point dose response with 3-fold dilutions (0.001–30 µM) as more potent compounds were identified. After 1 h the plate was re-spun and cells co-dosed with different concentrations of MMS (50–250 µg/mL final concentration) or DMSO using the Echo 550 and incubated for a given time (30 min–2 h) at 37°C in a cell culture incubator. Media was removed from the plate by inversion and cells were fixed with ice-cold 95% methanol/ phosphate buffered saline (PBS) for 15 min at -20°C and then washed once with PBS at room temperature. Cells were then permeabilized using PBS/Triton 0.1% for 20 min, and washed once in PBS before adding anti-PAR antibody (10H (#AM80), Merck Millipore) at 1:4000 in antibody blocking buffer (ADB; 5% Fetal bovine serum, 0.1% Tween20 in PBS) and incubated overnight at 4°C. Cells were then washed three times with PBS, before adding rabbit anti-mouse Alexofluor 488 (A11029, ThermoFisher) at 1:1000 and Hoechst 33342 (at 1:5000) in ADB and incubated for 1 h at room temperature and protected from light. Following three washes with PBS, the plates were sealed and images captured using a 10× objective on a CellInsight (ThermoFisher) and analysed using Cellomics Scan compartmental analysis software (ThermoFisher). A threshold determined by assessing the signal in DMSO treated cells was applied to the pixel intensity and a Box Detection application was used to detect objects smaller than five pixels in radius within the nucleus. The mean of the intensity of these nuclear spots at 488 nM or the mean intensity of total nuclear signal at 488 nM was reported. Initial assays shown in the Supplementary data used only a single dose of MMS for 0–60 min. Studies using temozolomide used the same procedure as with MMS, with a stock solution of temolozomide made at 20 mg/mL in DMSO.

### SRB assay

Cells in 96-well plates were fixed with the addition of 100 µL ice cold 10% trichloroacetic acid to the media. After 1 h at 4°C, the cells were washed twice with PBS and left to dry. Once dry, 100 µL 0.2% sulforhodamine B (SRB) was add to each well and incubated for 15 min at room temperature. The cells were washed three times with 200 µL 1% acetic acid and then dried. To solubilise the remaining SRB, 200 µL 10 mM Tris pH10.5 was added to each well and the plate incubated with agitation for 10 min. Absorbance at 520 nM was measured on a plate reader (Biotek, Swindon, UK).

## Results

Raw data for Figure 2–Figure 6 in ‘An assay to measure poly(ADP ribose) glycohydrolase (PARG) activity in cells’ Click here for additional data file. Copyright: © 2016 James DI et al. 2016 Data associated with the article are available under the terms of the Creative Commons Zero "No rights reserved" data waiver (CC0 1.0 Public domain dedication).

PARylation is principally driven by PARPs 1–3 after DNA damage and alkylating agents are known to induce base excision repair (BER) pathways, intermediates of which lead to activation of PARPs ^12^. Our preliminary data showed Hela cells that have been stably knocked down (KD) for PARG were more sensitive to growth inhibition by the alkylating agent MMS ( Supplemental Figure 1a). This led to the initial finding that 250 µg/mL MMS induced PAR chains in PARG KD cells and the peak of PAR chains detected was approximately 20 min after MMS addition ( Supplemental Figures 1b–d).

**Figure 1.**
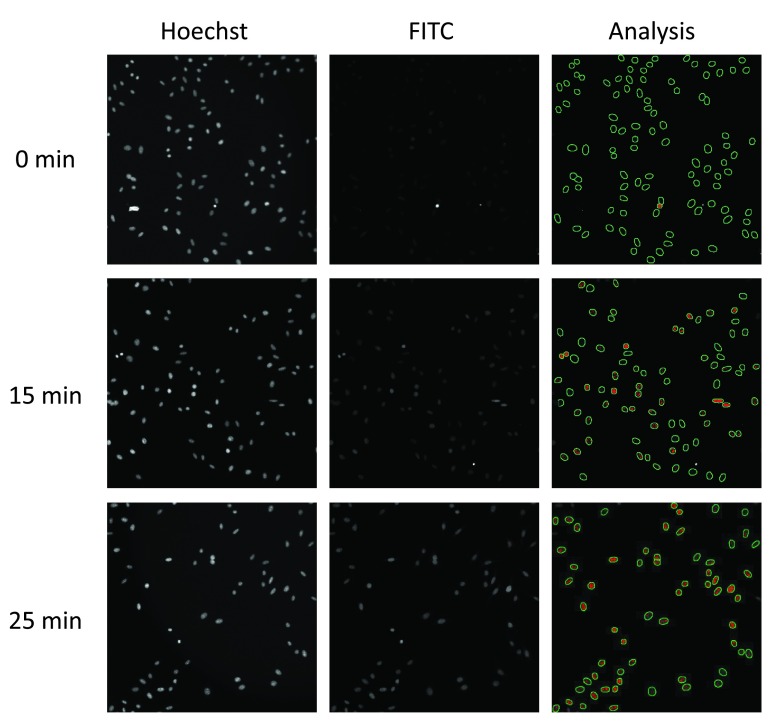
Hela cells treated with 250 µg/mL MMS showing increase in nuclear PAR signal. Using a high content imaging system the Hoechst stained nuclei (left-hand panels) are used to create a nuclear mask (green circle in Analysis). Anti-PAR antibody (FITC) detects the increase in PAR chains (centre panels) that is then quantified using the nuclear mask from the Hoechst signal (right-hand panels). Fluorescence intensity is shown as red dots within the nuclear mask.

Using the same antibody, an immunofluorescence assay was designed to detect PAR chains in cells. Hela cells were used as they showed increased PAR by western blot after MMS and responded to PARG KD by substantially increasing PAR after MMS ( Supplemental Figure 1b). We set up a standard assay based on our previous experience and online protocols for nuclear antigen detection. This used 95% methanol/PBS for fixation and 0.1% Triton X-100 for permeabilization. Hela cells were dosed with 250 µg/mL MMS for different amounts of time. Initial analysis of the PAR signal showed an increase in signal at approximately 25 min ( Figure 1). A nuclear mask was generated from Hoechst-stained cells to select regions of interest (ROI) in the 488 nm channel ( Figure 1 – analysis panels).

**Figure 2.**
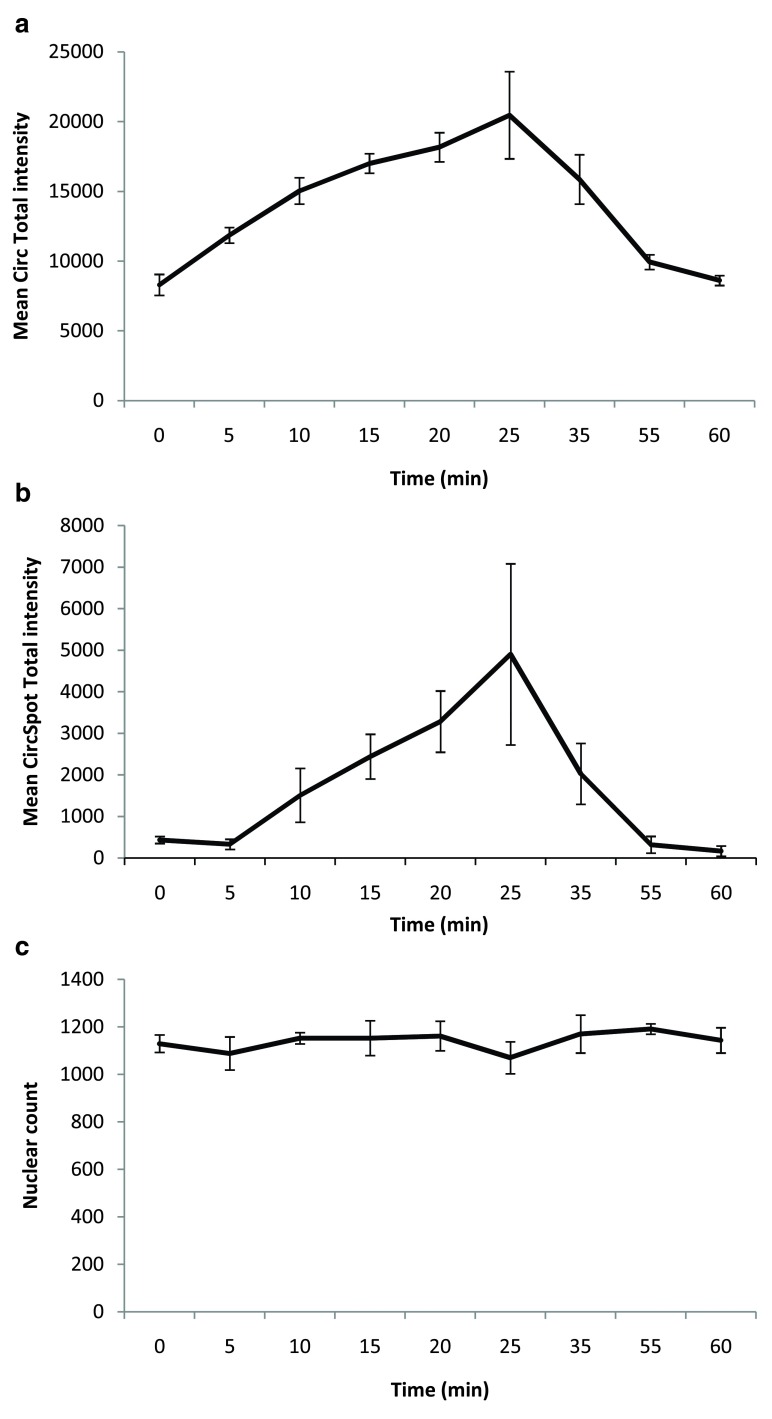
Parameter selection for nuclear PAR chains. ( **a**) The cellular average (from 9 fields) of the total intensity of nuclear fluorescence of PAR after 250 µg/mL MMS as a function of time. ( **b**) The cellular average (from 9 fields) of the total intensity of punctate nuclear fluorescence of PAR after 250 µg/mL MMS as a function of time. ( **c**) Analysis of cell number using Hoechst-stained nuclei after dosing with MMS showing that there is no decrease in total cell number after 1 h treatment.

Different parameters were selected on the Cellomics’ Scan software to report the intensity of the signal within the nuclear area (mask). Both the total intensity of the nuclear PAR signal ( Figure 2a; mean_circtotalintensity) and the total intensity of PAR signal points (spots) within the nucleus ( Figure 2b; mean_circspottotalintensity) showed a maximum at 25 min and then returned to baseline after 60 min. However, the total intensity of nuclear spots was chosen as the parameter for ongoing experiments as this provided the greatest signal window. We also noted that there was no significant change in cell number over the time course of the experiment ( Figure 2c).

**Figure 3.**
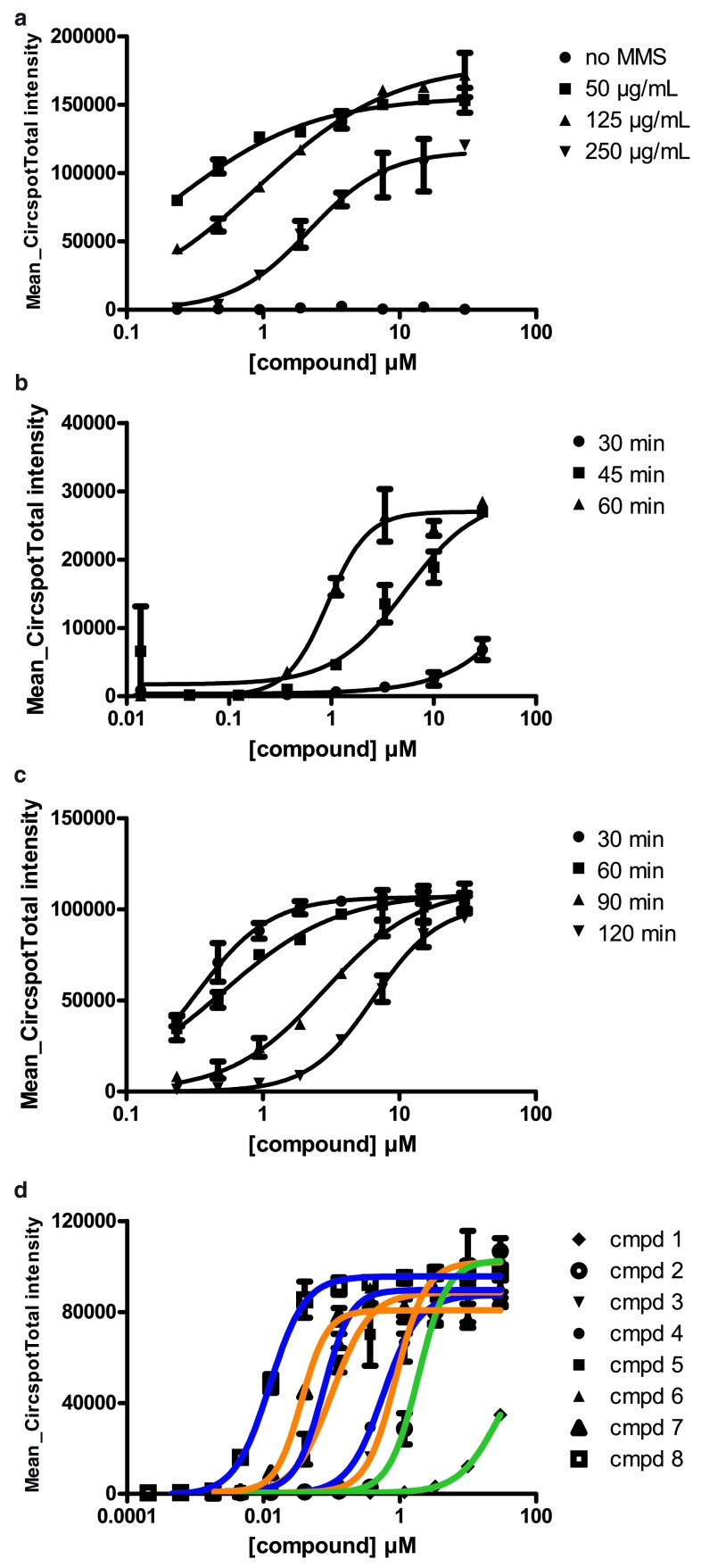
Enhancing PAR chain assay sensitivity. ( **a**) Decreasing the concentration of MMS moved the PARG inhibitor IC _50_ to the left indicating a greater sensitivity. ( **b**) PAR signal response with 25 µg/mL MMS shows that lower doses of MMS only elicit a nuclear PAR response with longer incubation times. ( **c**) Increasing the time of incubation with 50 µg/mL MMS shifts the PARG inhibitor IC _50_ curve to the right decreasing sensitivity. ( **d**) A selection of eight PARG inhibitor compounds from a PARG biochemical screen with a range of potencies also shows a range of sensitivities with this PAR chain assay. Different chemical cores of the compounds are shown (green, orange, blue). The compounds are ordered by sensitivity (cmpd 1, least sensitive; cmpd 8, most sensitive). Compound 4 is PDD00016133.

We initiated a drug discovery programme into PARG inhibitors based on the results of a high throughput screening (HTS) assay of 1.4M compounds ^11^. Using a prototype PARG inhibitor from this programme (PDD00016133) we tested a dose response with 0–250 µg/mL MMS ( Figure 3a) and 1 h of incubation post MMS dosing. This time point was chosen because at this time, in the absence of PARG inhibition, PAR chain detection has returned to base level. Pleasingly, DMSO alone (no MMS) had no measureable effect on nuclear PAR chains ( Figure 3a). However, PDD00016133 gave a dose-dependent increase in nuclear PAR signal in MMS-treated cells. In our biochemical assay, the same compound gave an EC _50_ of 0.36 µM (n=22) and we were surprised that the apparent cellular EC _50_ 2.2 µM was significantly less potent. We therefore tested lower concentrations of MMS and showed that decreasing MMS to 50 µg/mL increased the sensitivity of the assay and indicated that further dilutions of the compound needed to be made to generate a full EC _50_ curve ( Figure 3a).

Decreasing the concentration of alkylating agent clearly changed the observed PAR chain response although too little MMS decreased sensitivity ( Figure 3b). We therefore investigated how the PAR chain signal changed with time after dosing with 50 µg/mL MMS ( Figure 3c). Two hours of exposure to 50 µg/mL MMS provided a dose-response to PARG inhibition with PDD00016133, but with EC _50_ values increased (6.7 µM) when compared to high doses of MMS seen in Figure 3a. Decreasing the incubation time with 50 µg/mL MMS moved the dose response curve to the left with 30–60 min showing the best response (EC _50_ = 0.3 µM and 0.5 µM respectively). However, in both of these shorter incubation times we still observed high levels of nuclear PAR signal at the lowest dose of the PARG inhibitor. We therefore increased the dose range and tested a 10-point dose response with 3-fold dilutions between each point. A 1 h incubation time was chosen as this provided optimum sensitivity as well as enough time to dose and process a large number of plates. These assay conditions were tested with a selection of PARG inhibitors with different sensitivities from our biochemical assay. The combination of a 10-point dose response of the PARG inhibitor with 50 µg/mL MMS for 1 h clearly demonstrated that we had cell permeable inhibitors of PARG that ranged from low nanomolar to micromolar potencies ( Figure 3d).

**Figure 4.**
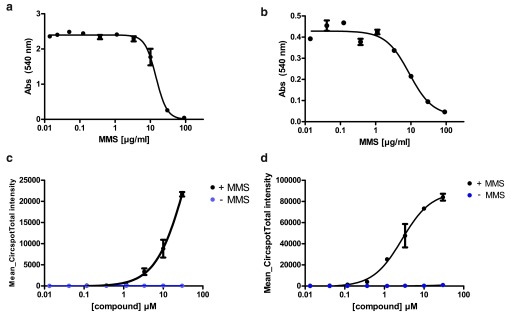
PAR chain detection in different cell lines. ( **a**) MEFs and ( **b**) SCLC H1048 treated with MMS were fixed and stained with sulforhodamine B (SRB) after 72 h. ( **c**) MEFs and ( **d**) H1048 cells show a dose-dependent increase in PAR chains after MMS treatment.

We then explored whether other cell lines or other DNA damaging agents could be used with this assay. Firstly, we explored if murine cells responded to MMS. Murine embryonic fibroblasts (MEFs) and the human small cell lung cancer cell line H1048 were dosed with MMS and showed a similar IC _50_ compared with Hela cells (14.5 µM and 9.0 µM, Figure 4a, b). The PAR chain assay was run on MEFs with the inhibitor PDD00016133 and 50 µg/mL MMS for 1 h. In the absence of MMS there was no increase in nuclear PAR chains detected with this inhibitor. However, in the presence of MMS, the PARG inhibitor led to a dose-dependent increase in nuclear PAR chain signal ( Figure 4c). This dose-dependent increase in PAR chain signal after MMS was also seen in H1048 cells ( Figure 4d).

**Figure 5.**
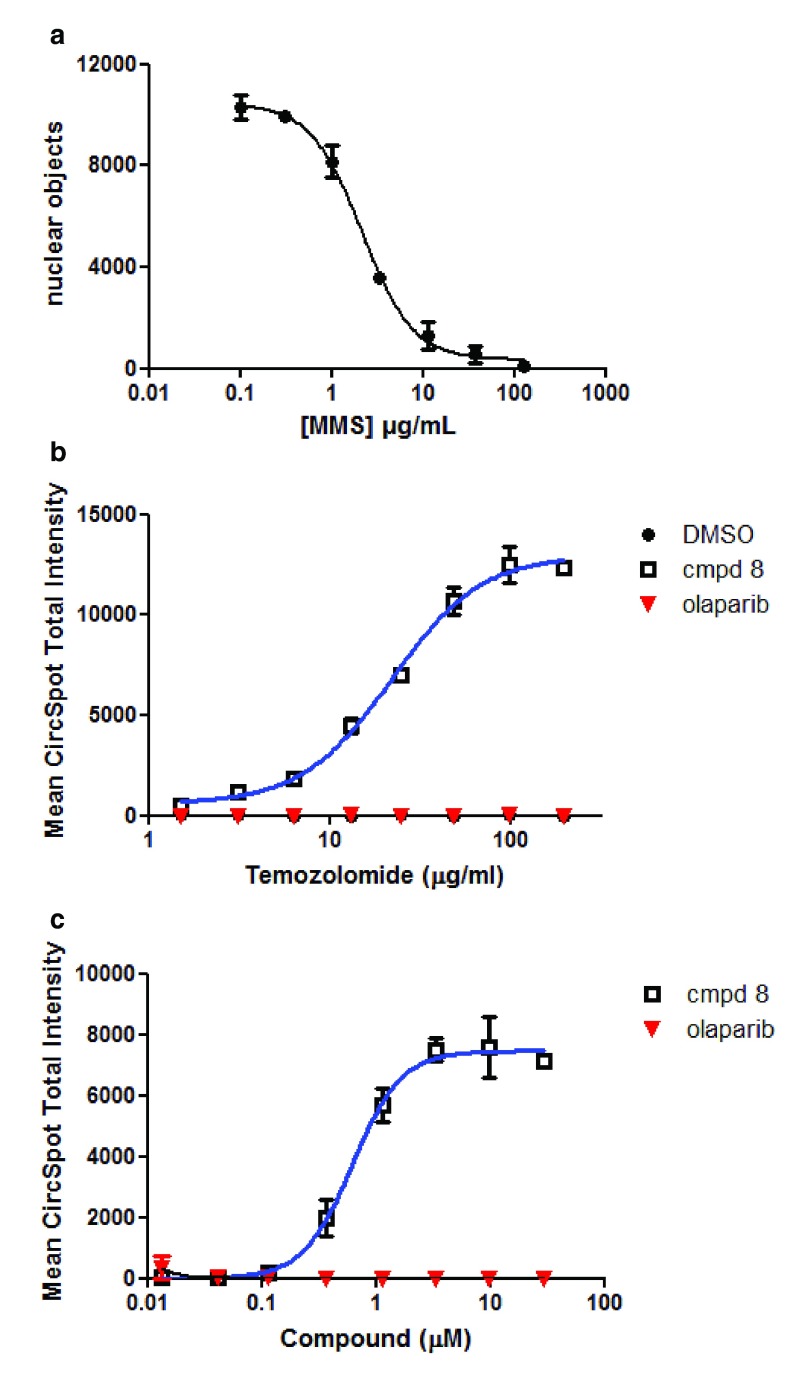
Response of SW620 colorectal cancer cells to alkylating agents. ( **a**) SW620 cells treated with MMS for 72 h and stained with Hoechst show a similar dose-dependent decrease in proliferation in comparison with other cell lines tested ( **b**) SW620 cells pre-treated with compound 8 at 300 nM increase PAR chains in response to 1 h temozolomide (1.5 µg/mL–200 µg/mL). However, pre-treatment with DMSO or olaparib (300 nM) had no effect on PAR chains at this time point. ( **c**) SW620 cells treated with increasing concentrations of a PARG inhibitor (cmpd 8) and 150 µg/mL temozolomide for 1 h showed a dose-dependent increase in PAR chains. As expected at this time point treatment with olaparib had no effect on PAR chains.

We next explored whether a more clinically relevant DNA alkylating agent could induce PAR chains. Temozolomide (TMZ) is a DNA alkylating agent and is used as a standard-of-care treatment for patients with glioblastoma ^13^. Here we used the colorectal cancer cell line SW620 that we knew was sensitive to alkylating agents ( Figure 5a) and which has been used in xenograft studies in combination with TMZ and the PARP inhibitors olaparib or AG014699 ^14, 15^. First we used the same assay conditions to determine whether increasing concentrations of TMZ induced PAR chains that could be maintained by inhibiting PARG with a potent inhibitor (compound 8 from Figure 3d). As expected from the previous cell lines, one hour after treatment with TMZ alone there was no PAR signal detectable in SW620 cells. However, the presence of 300 nM compound 8 led to a TMZ dose-dependent increase in PAR signal ( Figure 5b). Furthermore, using a set amount of TMZ (150 µg/mL) we were able to show that PARG inhibition by compound 8 led to a dose-dependent increase in PAR signal ( Figure 5c). Unsurprisingly, pre-treatment with olaparib, which prevents PARP1 PARylation did not lead to any increase in PAR signal after TMZ treatment.

**Figure 6.**
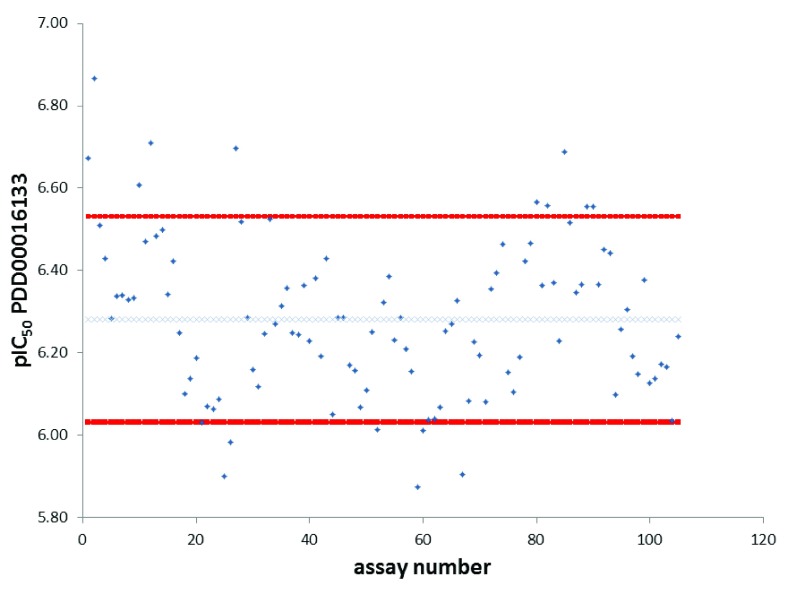
PAR chain assay robustness over time. The PAR assay was run over a period of 2½ years resulting in over 100 assays. The pIC _50_ for compound PDD00016133 was plotted for each assay. The pIC _50_ geomean is indicated with the blue crossed line and *±* 0.25 pIC _50_ is indicated with the red lines.

Finally, we quantified the relationship between individual assay results in Hela cells for PDD00016133 against its geomean over a period of 2½ years ( Figure 6). Over 100 assays with PDD00016133 were run during that time, of which 85% were within ±0.25×pIC _50_ of its geomean. Interestingly, cell cultures that had passage numbers of less than 8 or more than 19 were more likely to give results for this compound that exceeded these limits.

## Discussion

A number of molecules have been used to inhibit PARG but concerns have been raised as to their selectivity and potency both in biochemical assays and in cells. As part of a drug discovery programme for PARG inhibitors we designed and optimized a cell assay for PARG inhibitor activity. Our initial work showed that the higher dose of MMS (250 µg/mL) resulted in a complete dose response curve for our PARG inhibitor but potency was lower than we expected. By reducing the amount of DNA damage the sensitivity of the assay increased, presumably as the detection of SSBs by PARP1 and its associated machinery was not overwhelmed. However, the lowest dose of the PARG inhibitor still resulted in relatively high levels of PAR chains after 30–60 min that was resolved when the dose response was extended.

Immunofluorescence assays using the 10H mouse hybridoma antibody for detecting PAR were first published over 20 years ago ^16^. However, detailed quantification using immunofluorescence of the amount of PAR chains found after DNA damage appears to be absent from the literature. Instead, enzyme linked immune absorbance assays (ELISA) or dot-blots have been used to detect the reduction of PAR chains following the use of PARP inhibitors ^17– 19^. There have been studies that have followed the kinetics of PAR chain accumulation after treatment with the alkylating agent MNNG or the oxidant H _2_O _2_^20^ but none on the increase of PAR following temozolomide treatment. However, studies using RNA interference have been able to show a delay in hydrolysis of nuclear PAR after treatment with H _2_O _2_ and knockdown of PARG ^21^.

The suitability of this assay for screening PARG inhibitors in Hela cells is clear from the data collected over time and with different compounds ( Figure 3d and Figure 6). However, MEFs and H1048 cells displayed a response that was indicative of the Hela cell response prior to optimisation ( Figure 4c, d), suggesting that more method development would be needed if these cells were going to be used for routine testing.

This assay was designed to test for PARG inhibition after a DNA damage signal. However, a number of PARPs are involved in non-DNA damage related processes (e.g. tankyrases, reviewed in 22) that take place outside the nucleus. Hydrolysis of PAR chains created by other PARPs is likely to involve PARG or ARH3 ^23^. It is possible that these PARG inhibitors prevent such processes but modification of this assay would have to be undertaken to detect non-nuclear PAR.

In summary, we have designed a sensitive assay to test for PARG inhibition in cells. The assay was appropriate and stable for long term use and detected PAR chains from different species and different cell lines.

## Data availability

The data referenced by this article are under copyright with the following copyright statement: Copyright: © 2016 James DI et al.

Data associated with the article are available under the terms of the Creative Commons Zero "No rights reserved" data waiver (CC0 1.0 Public domain dedication). 

*F1000Research*: Dataset 1. Raw data for Figure 2– Figure 6 in ‘An assay to measure poly(ADP ribose) glycohydrolase (PARG) activity in cells’, 10.5256/f1000research.8463.d119225 ^24^

## Supplementary material

Figure 1. Preliminary results during assay development. ( **a**) PARG KD Hela cells (PARG Silencix) are more sensitive to MMS than control cells (IC _50_ 6.2 µg/mL *vs* 16.5 µg/mL) over 5 days. ( **b**) PAR detection by western blot shows that MMS induced PAR response in control (-) and a substantial response in PARG KD cells (KD). ( **c**) HT29 cells treated with 250 µg/mL MMS show an initial increase in PAR signal at 20 min with a gradual reduction in detectable PAR chains. ( **d**) Cartoon of proposed PAR chain response in cells.

Supplementary methods and notes on compounds used.

## References

[1] Alvarez-Gonzalez R Jacobson MK: Characterization of polymers of adenosine diphosphate ribose generated *in vitro* and *in vivo*. *Biochemistry.* 1987;26(11):3218–24. 10.1021/bi00385a042 3038179

[2] Kelley MR Logsdon D Fishel ML: Targeting DNA repair pathways for cancer treatment: what's new? *Future Oncol.* 2014;10(7):1215–37. 10.2217/fon.14.60 24947262 PMC4125008

[3] Frampton JE: Olaparib: a review of its use as maintenance therapy in patients with ovarian cancer. *BioDrugs.* 2015;29(2):143–50. 10.1007/s40259-015-0125-6 25899311

[4] Bakondi E Bai P Erdélyi K: Cytoprotective effect of gallotannin in oxidatively stressed HaCaT keratinocytes: the role of poly(ADP-ribose) metabolism. *Exp Dermatol.* 2004;13(3):170–8. 10.1111/j.0906-6705.2004.0150.x 14987257

[5] Okita N Ashizawa D Ohta R: Discovery of novel poly(ADP-ribose) glycohydrolase inhibitors by a quantitative assay system using dot-blot with anti-poly(ADP-ribose). *Biochem Biophys Res Commun.* 2010;392(4):485–9. 10.1016/j.bbrc.2010.01.044 20079708

[6] Slama JT Aboul-Ela N Jacobson MK: Mechanism of inhibition of poly(ADP-ribose) glycohydrolase by adenosine diphosphate (hydroxymethyl)pyrrolidinediol. *J Med Chem.* 1995;38(21):4332–6. 10.1021/jm00021a023 7473561

[7] Finch KE Knezevic CE Nottbohm AC: Selective small molecule inhibition of poly(ADP-ribose) glycohydrolase (PARG). *ACS Chem Biol.* 2012;7(3):563–70. 10.1021/cb200506t 22220926 PMC3306470

[8] Steffen JD Coyle DL Damodaran K: Discovery and structure-activity relationships of modified salicylanilides as cell permeable inhibitors of poly(ADP-ribose) glycohydrolase (PARG). *J Med Chem.* 2011;54(15):5403–13. 10.1021/jm200325s 21692479 PMC3150619

[9] Dunstan MS Barkauskaite E Lafite P: Structure and mechanism of a canonical poly(ADP-ribose) glycohydrolase. *Nat Commun.* 2012;3: 878. 10.1038/ncomms1889 22673905

[10] Formentini L Arapistas P Pittelli M: Mono-galloyl glucose derivatives are potent poly(ADP-ribose) glycohydrolase (PARG) inhibitors and partially reduce PARP-1-dependent cell death. *Br J Pharmacol.* 2008;155(8):1235–49. 10.1038/bjp.2008.370 18806807 PMC2607208

[11] Stowell AI James DI Waddell ID: An HTS-compatible HTRF assay measuring the glycohydrolase activity of human PARG. *Anal Biochem.*in press.2016; S0003-2697(16)00116-0. 10.1016/j.ab.2016.03.016 27036617

[12] Horton JK Stefanick DF Prasad R: Base excision repair defects invoke hypersensitivity to PARP inhibition. *Mol Cancer Res.* 2014;12(8):1128–39. 10.1158/1541-7786.MCR-13-0502 24770870 PMC4135006

[13] Hottinger AF Stupp R Homicsko K: Standards of care and novel approaches in the management of glioblastoma multiforme. *Chin J Cancer.* 2014;33(1):32–9. 10.5732/cjc.013.10207 24384238 PMC3905088

[14] Thomas HD Calabrese CR Batey MA: Preclinical selection of a novel poly(ADP-ribose) polymerase inhibitor for clinical trial. *Mol Cancer Ther.* 2007;6(3):945–56. 10.1158/1535-7163.MCT-06-0552 17363489

[15] Ali M Kamjoo M Thomas HD: The clinically active PARP inhibitor AG014699 ameliorates cardiotoxicity but does not enhance the efficacy of doxorubicin, despite improving tumor perfusion and radiation response in mice. *Mol Cancer Ther.* 2011;10(12):2320–9. 10.1158/1535-7163.MCT-11-0356 21926192 PMC3242069

[16] Bürkle A Chen G Küpper JH: Increased poly(ADP-ribosyl)ation in intact cells by cisplatin treatment. *Carcinogenesis.* 1993;14(4):559–61. 10.1093/carcin/14.4.559 8472314

[17] Plummer ER Middleton MR Jones C: Temozolomide pharmacodynamics in patients with metastatic melanoma: dna damage and activity of repair enzymes *O* ^6^-alkylguanine alkyltransferase and poly(ADP-ribose) polymerase-1. *Clin Cancer Res.* 2005;11(9):3402–9. 10.1158/1078-0432.CCR-04-2353 15867241

[18] Liu X Palma J Kinders R: An enzyme-linked immunosorbent poly(ADP-ribose) polymerase biomarker assay for clinical trials of PARP inhibitors. *Anal Biochem.* 2008;381(2):240–7. 10.1016/j.ab.2008.07.007 18674509

[19] Ida C Yamashita S Tsukada M: An enzyme-linked immunosorbent assay-based system for determining the physiological level of poly(ADP-ribose) in cultured cells. *Anal Biochem.* 2016;494:76–81. 10.1016/j.ab.2015.10.014 26548958 PMC6118347

[20] Cortes U Tong WM Coyle DL: Depletion of the 110-kilodalton isoform of poly(ADP-ribose) glycohydrolase increases sensitivity to genotoxic and endotoxic stress in mice. *Mol Cell Biol.* 2004;24(16):7163–78. 10.1128/MCB.24.16.7163-7178.2004 15282315 PMC479728

[21] Blenn C Althaus FR Malanga M: Poly(ADP-ribose) glycohydrolase silencing protects against H _2_O _2_-induced cell death. *Biochem J.* 2006;396(3):419–29. 10.1042/BJ20051696 16526943 PMC1482814

[22] Haikarainen T Krauss S Lehtio L: Tankyrases: structure, function and therapeutic implications in cancer. *Curr Pharm Des.* 2014;20(41):6472–88. 10.2174/1381612820666140630101525 24975604 PMC4262938

[23] Oka S Kato J Moss J: Identification and characterization of a mammalian 39-kDa poly(ADP-ribose) glycohydrolase. *J Biol Chem.* 2006;281(2):705–13. 10.1074/jbc.M510290200 16278211

[24] James D Durant S Eckersley K: Dataset 1 in: An assay to measure poly(ADP ribose) glycohydrolase (PARG) activity in cells. *F1000Research.* 2016 Data Source 10.12688/f1000research.8463.1 PMC4995692 27610220

