# Supplementary figures and images for: An assay to measure poly(ADP ribose) glycohydrolase (PARG) activity in cells
Source: F1000Res. 2016 Sep 15;5:736. Originally published 2016 Apr 25. [Version 2] doi: 10.12688/f1000research.8463.2 (PMC4995692; doi:10.12688/f1000research.8463.2)

## Slide 1
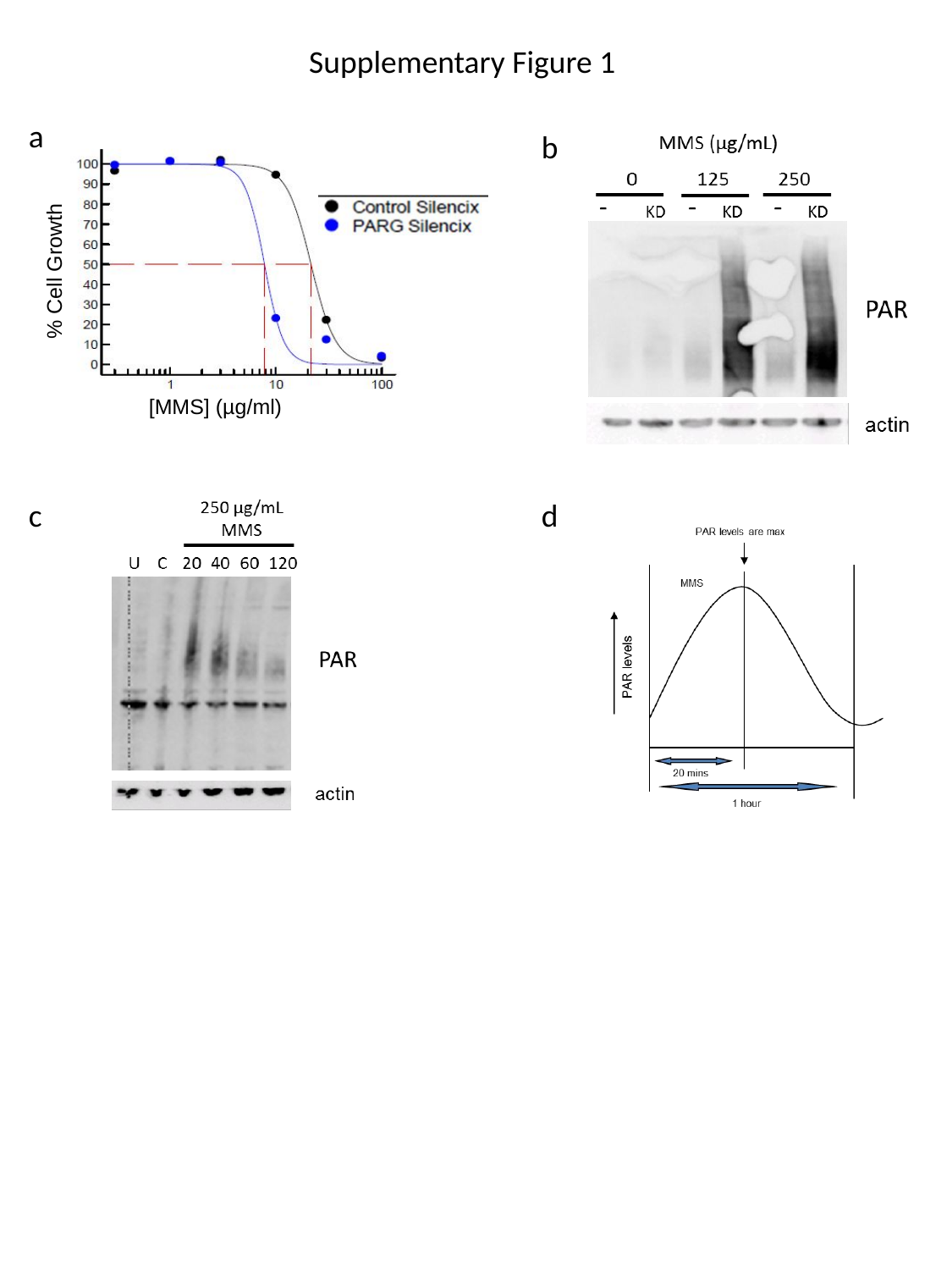

Supplementary Figure 1
a
b
% Cell Growth
[MMS] (µg/ml)
c
d

Supplement: Supplementary file 2 [file f1000research-5-10347-s0001.tgz › 7e73f4c9-b89a-4f24-8e65-9f4578894a23.pptx]
