# Supplementary material for: An assay to measure poly(ADP ribose) glycohydrolase (PARG) activity in cells
Source: F1000Res. 2016 Sep 15;5:736. Originally published 2016 Apr 25. [Version 2] doi: 10.12688/f1000research.8463.2 (PMC4995692; doi:10.12688/f1000research.8463.2)
Supplement: Supplementary file 3 [file f1000research-5-10347-s0002.tgz › 2d571439-9cd0-43a3-a5e0-48f6a044c22a.docx]

**An assay to measure poly(ADP ribose) glycohydrolase (PARG) activity in cells**

Dominic I James^1,*^ Stephen Durant^2^, Kay Eckersley^2^, Emma Fairweather^1^, Louise A Griffiths^1^, Nicola Hamilton^1^, Paul Kelly^1^, Mark O’Connor^2^, Kerry Shea^2^, Ian D Waddell^1^ and Donald J Ogilvie^1^.

^1^ Drug Discovery Unit, Cancer Research UK Manchester Institute, University of Manchester, Wilmslow Road, Manchester, M20 4BX, U.K.

^2^ Oncology iMED, AstraZeneca Pharmaceuticals, Alderley Park, Macclesfield, Cheshire, SK10 4TG, U.K.

* Corresponding author. E-mail: [dominic.james@cruk.manchester.ac.uk](mailto:dominic.james@cruk.manchester.ac.uk)

Supplementary methods

Western blot

Cells were seeded in 6-well plates and the following day treated with DMSO, 125 µg/mL, 250 µg/mL MMS for 20 min. Media was then aspirated and cells gently washed with 1 mL ice-cold PBS. To each well, 75 µL lysis buffer (25 mM Tris-HCl, 3 mM EDTA, 3 mM EGTA, 50 mM NaF, 2 mM Na_3_VO_4_ , 0.27 mM sucrose, 10 mM β-glycerophosphate, 5 mM sodium pyrophosphate, 2 % Triton X-100) with complete protease inhibitor (Roche) and 5 µM APD-HPD was added and incubated for 10 min on ice. Lysates were transferred to eppendorf tubes containing 30 µL 4x sample buffer (Biorad, Hemel Hempstead, UK) and incubated for 5 min at 95°C. Samples were then loaded on a 3–8% tris-acetate gel and following separation transferred to nitrocellulose. The membrane was blocked with 10% non-fat milk powder in tris-buffered saline (TBS) + 0.05% Tween20 (TBST) for 1 h. Mouse anti-PAR antibody (#550781, BD Pharmingen, Oxford, UK) was incubated overnight at 4°C. After washing with TBST, the membrane was incubated with anti-mouse secondary (Abcam) 1:5000 in 5% BSA for 1 h. After washes with TBST the antibody complexes were detected with ECL. After washing with Restore (ThermoFisher) according to manufacturer’s recommendations, the membrane was reprobed as above but using mouse actin antibody (Sigma #A5441) at 1:5000 in 5% non-fat dried milk powder in TBST. For HT29 westerns, cells were treated with 250 µg/mL MMS for the times shown and PAR detected using anti-PAR clone 10H (Merck Millipore) using the procedure outlined above.

Supplementary notes

Compounds in Figure 3d are from CRUK MI PARG drug discovery programme and their numbers are as follows: cmpd 1 = PDD00013907, cmpd 2 = PDD00014104, cmpd 3 = PDD00015009, cmpd 4 = PDD00016133, cmpd 5 = PDD00016845, cmpd 6 = PDD00016882, cmpd 7 = PDD00017155, cmpd 8 = PDD00017272.
