# Supplementary material for: An assay to measure poly(ADP ribose) glycohydrolase (PARG) activity in cells
Source: F1000Res. 2016 Sep 15;5:736. Originally published 2016 Apr 25. [Version 2] doi: 10.12688/f1000research.8463.2 (PMC4995692; doi:10.12688/f1000research.8463.2)
Supplement: Peer review history

# Peer review: Referee response for version 1

**DOI:** 10.5256/f1000research.9113.r15536

**Published:** 2016-08-19

**Authors:** Dik C van Gent, Titia Meijer

**Affiliations:** 1 Department of Molecular Genetics, Erasmus University Medical Center, Rotterdam, Netherlands; 2 Department of Pathology, Erasmus University Medical Center, Rotterdam, Netherlands

This manuscript describes a useful and robust method to quantify PAR chains in cells after various treatments. This is important for evaluating the effects of various PARP and PARG inhibitors. Results and conclusions are in general well described and appropriate.

 I have some minor comments, mainly about the text and figure legends:

 The Cellomics’ Scan software was used to report the intensity of the PAR signal within the nuclear area, either quantified as the total intensity of the nuclear PAR signal or the total intensity of PAR signal points within the nucleus. Could the author clarify what defines a signal point? It would be useful for researchers using other software to reproduce these findings. Furthermore, could the authors indicate whether another method, such as simple thresholding, would also be possible? The curves in figures 3a and b suggest that the result might be quite similar.

- Figure 1: Last sentence ‘in’ should be removed.
- Figure 3: The text describing figure 3a and 3b in the legend was switched. Also, I would indicate in the figure that in 3c 50ug/ml MMS was used, as the concentration MMS is indicated for 3a and 3b but not for 3c. Also the sentence on page 6, lines 4-8, is not entirely clear: ‘… increase the sensitivity of the assay but the bottom of the curve was not reached.’ Is a somewhat difficult way to say that the authors used a range that did not go to low enough concentrations to reach background levels. Probably this sentence could be adapted for easier understanding.
- Figure 3d: use different colors for each different compound. It is now very hard to distinguish which line represents which compound.
- Figure 5a: after 72 the word ‘hours’ should be added.

We have read this submission. We believe that we have an appropriate level of expertise to confirm that it is of an acceptable scientific standard.

# Response

**Published:** 2016-09-08

**Authors:** Dominic James

**Affiliations:** Cancer Research UK Manchester Institute, UK

Many thanks for taking the time to carefully read this manuscript and for your very detailed comments. We have addressed the minor typos. In addition, we have added the following to the method text:

 “and analysed using Cellomics Scan compartmental analysis software (ThermoFisher). A threshold determined by assessing the signal in DMSO treated cells was applied to the pixel intensity and a Box Detection application was used to detect objects smaller than five pixels in radius within the nucleus. The mean of the intensity of these nuclear spots at 488 nM or the mean intensity of the total nuclear signal at 488 nM was reported.”

 Simple thresholding, if applied to the nuclear area alone, would also achieve similar results and was initially carried out using ImageJ on images obtained from a microscope before the CellInsight was purchased.

 To address the slightly odd wording regarding the results from Figure 3, we have changed the sentence to read, “Increased the sensitivity of the assay and indicated that further dilutions of the compound needed to be made to generate a full EC _50_ curve.”. The point we were trying to convey was that a good screening assay should generate the sigmoidal curves with data points on all parts of the curve.

 Regarding Figure 3d, unfortunately it is not possible to only change the colours next to the compound names. In Prism, the points on the graph would also end up in different colours, which we felt was unappealing to look at. The main point which this figure is mean to convey is that we have used the assay to assess a number of compounds that have been shown to have very different potencies by a biochemical assay and this cell assay also discriminates between them. To aid visualisation we have added the sentence “The compounds are ordered by sensitivity (cmpd 1, least sensitive; cmpd 8, most sensitive)”.

# Peer review: Referee response for version 1

**DOI:** 10.5256/f1000research.9113.r14957

**Published:** 2016-07-21

**Authors:** Xiaochun Yu

**Affiliations:** 1 Department of Cancer Genetics and Epigenetics, Beckman Research Institute, City of Hope Medical Center, Duarte, CA, USA

Poly(ADP-ribosyl)ation is activated in response to DNA damage. In this manuscript, the authors used the immunostaining assay to examine the DNA damage-induced PAR chain and measured the dePARylation process in cells. The assay itself is very simple and reliable. However, besides PARG, there are other dePARylation enzymes contributing for the cellular dePARylation. And the list of the dePARylation enzymes is still growing. Since this assay is to measure overall dePARylation instead of specifically examining the PARG activity, the authors are suggested to modify the title of this manuscript, which might be better to convey their message.

I have read this submission. I believe that I have an appropriate level of expertise to confirm that it is of an acceptable scientific standard.

# Response

**Published:** 2016-09-08

**Authors:** Dominic James

**Affiliations:** Cancer Research UK Manchester Institute, UK

Many thanks for taking the time to carefully read this manuscript and for your helpful comment regarding other dePARylating enzymes.

 You are indeed correct as recent in vitro evidence has shown that PAR chains can be cleaved by phosphodiesterases (NUDT16, ENPP1) but their involvement in the hydrolysis of PAR chains after DNA damage in cells has not been shown. The PARG inhibitors are therefore most likely inhibiting PAR chain hydrolysis by PARG but we cannot rule out that other, as yet unidentified, proteins involved in the de-PARylation may well exist that may be affected by our PARG inhibitors. However, our biochemical assay (Stowell et al. reference 11) does show the selectivity of our compounds for PARG over PARP and ARH3 and another submitted manuscript details the binding of the inhibitors to PARG by crystallography and surface plasmon resonance (SPR).

 I think on reflection that we should keep the initial title of the manuscript.
